# A warming-yang herbal decoction protects against high-altitude hypoxia–induced osteoblast injury through modulation of HIF-1 signaling

**DOI:** 10.3389/fphar.2025.1673146

**Published:** 2025-12-18

**Authors:** Xuehai Liu, Yaqian Qi, Rui Zhao, Binkui Jia, Xilin Lei, Hongfan Li, Di Wang, Haoling Zhang, Wei Wang, Zhijing Song

**Affiliations:** 1 Clinical College of Traditional Chinese Medicine, Gansu University of Chinese Medicine, Lanzhou, China; 2 Department of Biomedical Sciences, Advanced Medical and Dental Institute, Universiti Sains Malaysia, Minden Heights, Penang, Malaysia; 3 College of Acupuncture-Moxibustion and Tuina, Gansu University of Chinese Medicine, Gansu University of Chinese Medicine, Lanzhou, Gansu, China; 4 Dunhuang Key Laboratory of Medicine and Transformation, Ministry of Education, Lanzhou, China

**Keywords:** high-altitude environment, osteoporosis, MC3T3-E1 cells, HIF-1 signaling pathway, warming yang medicine

## Abstract

**Objective:**

This paper systematically explored dental osteoblast injury at high altitude hypoxic condition and examined the protective role of warming-yang traditional medicine, Aconitum carm michaelii and Zingiber officinale decoction (GAWD) in this condition.

**Methods:**

We constructed an *in-situ* high-altitude hypoxic model (4,500 m) with MC3T3-E1 osteoblastic cells and conducted RNA-seq analysis for Differentially Expressed Genes (DEGs). We performed enrichment analyses (GO, KEGG, and the Reactome pathway mapping) to reveal the relationships among the DEGs and key pathways. To further verify our results, we conducted a series of functional assays, including Cell Counting Kit-8 (CCK-8) for cell viability; flow cytometry assessment of reactive oxygen species (ROS); jc-10-based evaluation of mitochondrial membrane potential (MMP), and alkaline phosphatase (ALP) activity serum.

**Results:**

Transcriptome profiling revealed that the hypoxia-inducible factor-1 (HIF-1) signaling pathway was central in the mediator role of osteoblast injury. High altitude hypoxia greatly suppressed the expression of HIF-1α, HIF-1β, von Hippel–Lindau tumor suppressor gene (VHL) and vascular endothelial growth factor (VEGF), resulting in increased intracellular ROS, calcium overload, mitochondrial distress, G_1_-phase cell cycle arrest, apoptosis, and decreased proliferation. Rehabilitation by GAWD recovered these impairment effects: cell viability and apoptotic protein expression were improved, ROS and intracellular Ca^2+^ were reduced, and mitochondrial membrane potential was regained, HIF-1/VEGF axis was recovered, apoptotic signaling and gross morphological injury were attenuated, and overall osteoblastic function was restored.

**Conclusion:**

The findings implicated HIF-1 signaling dysregulation in inducing high-altitude hypoxia–induced osteoblast injury and osteoporosis. GAWD may reduce hypoxia–induced cellular stress through recovering redox homeostasis and adjusting HIF-1/VEGF signaling. Collectively, this, study offers early molecular evidence supporting the utility of GAWD as a traditional Chinese medicine–based intervention for the prevention of bone metabolic disorders due to chronic hypoxic exposure.

## 1 Introduction

Osteoporosis (OP) is a systemic skeletal disorder in which there is reduced bone mass and impairment of the bone microarchitecture (Słupski et al., 2021). Accumulating evidence suggests that mitochondrial dysfunction at the level of osteoblasts impairs energy metabolism and reduces bone formation, eventually leading to an imbalance between bone formation and resourcing, thus contributing to the progressive phenotype by which OP progresses (Zhang et al., 2025). Furthermore, ATP-induced cell death promotes osteoclastogenesis by stimulating the release of receptor activator of nuclear factor κB ligand (RANKL), a primary cytokine that encourages the differentiation of osteoclasts. This mechanism also induces apoptosis in osteocytes, further worsening bone loss (Wang J. et al., 2023).

As expanded human activity continues in high-altitude areas, over 520 million people now live above 1,500 m around the world. On the Tibetan Plateau, covering about a third of China’s total land area, over 60 million people form one of the largest high-altitude populations in the world (Zhang et al., 2024). Researchers have already shown that elevation gradually leads to poorer skeletal health and decreasing atmospheric oxygen gradually disrupts the dynamic equilibrium between osteoblast-mediated bone formation and osteoclast-mediated bone resorption under the progression of osteoporosis (Bozzini et al., 2013). Although osteoporosis in lowland populations arises mainly from the imbalance of bone formation through osteoblasts and bone resorption through osteoclasts, the underlying natural pattern remains unchanged at high altitude. However, upstream regulatory factors are different—longterm hypoxia, oxidative stress, and the dysregulation of HIF-1 signaling are dominating the disturbance of the mechanism to accelerate bone loss (Wang W. et al., 2023). Several types of environmental stress have been highlighted, especially the effects of low barometric pressure, hypoxia, and ultraviolet radiation; among them, hypobaric hypoxia and oxygen deficiency are known to be the main pathogenic factors that affect bone metabolism at high altitude (Camacho-Cardenosa et al., 2019; Da et al., 2021). The low atmospheric pressure and chronic hypoxia are main physiological stressors in high-altitude environments, likely leading to abnormal bone remodeling in animal and cellular models (Brent, 2022). Hypoxic conditions positively regulate bone metabolism through a dual role of HIF-1 signaling. While mild inductions of HIF-1 promote angiogenesis and osteogenesis, chronic hypoxia at high altitude activates the HIF-1 signaling network that unbalances the homeostasis of HIF-1, resulting in the dysfunction of mitochondrial metabolism, impaired bone marrowoblast proliferation, and impaired bone matrix biosynthesis. In turn, these changes are associated with osteogenic trauma and bone loss (Usategui-Martín et al., 2022). High altitude chronic hypoxia causes the excessive accumulation of ROS in osteoblasts, which consequently triggers the calcium overload in these cells, leading to the disruption ofANGIOGENATION-Osteogenesis coupling. The metabolic stress also downregulates the expression of VEGF via the instability of HIF-1α, hindering bone vascularization and reducing osteogenic potential (Chen et al., 2022; Yan et al., 2022).

HIF-1 is an essential regulatory channel to enable cell survival in glycolytic adaptation to low-oxygen conditions and has critical physiologies in oxygen regulation, glycolytic metabolism, angiogenesis, and mammary cell proliferation. HIF-1 is a heterodimer consisting of an oxygen-sensitive α-subunit (HIF-1α), and a constitutively expressed β-subunit (HIF-1β) (Bakleh and Al Haj Zen, 2025). Under normal oxygen levels, HIF-1α is hydroxylated by prolyl hydroxylases (PHDs), which target it to ubiquitination and proteasomal degradation through the VHL E3 ligase complex. Conversely, hypoxic conditions stabilize HIF-1α, which with HIF-1β forms a dimers to activate hypoxia-responsive genes, telles as VEGF (Bie et al., 2023). However, chronic or excessive activation of HIF-1ɑ has been confirmed to cause impaired osteoblast differentiation and disturbed bone mineralized (Qin et al., 2022). A prior study suggests that protracting hypoxia can lead to dysregulation of HIF-1α–VHL–VEGF signaling network and abnormal vascularization and altered bone metabolism (Zhao et al., 2012; Loots et al., 2018; Tremblay and Ainslie, 2021). Nevertheless, the oscillating regulation of this pathway in osteoblasts subjected to prolonged sustained high-altitude hypoxia is still poorly understood.

Pharmaceuticals for OP in Western medicine (e.g., bisphosphonates, calcitonin, and parathyroid hormone analogs) are clinically effective but sometimes constrained by side effects, such as gastrointestinal irritation, renal impairment, and even osteonecrosis of the jaw and unusual femoral fractures (Mikosińska et al., 2025). In contrast, traditional Chinese medicine (TCM), as a healing approach that replenishes metabolic activities and reversed cold-caused dysfunctions, uses “warming-Yang” agents for bone health. Aconitum carmichaelii Debeaux (treated as Fuzi) and Zingiber officinale Roscoe (dried ginger, Ganjiang) constitute the GAWD, which is the classic warming-Yang formula that restores Yang energetics, circulation, and metabolic suppression. Although TCM is considered a safer alternative that has multifaceted regulatory effects in comparison with western drugs, below, our focus is on the molecular mechanism of GAWD, and not on the direct pharmacological comparison between western drugs. A thorough analysis of its safety and efficacy compared to the traditional treatment will be checked out in future *in vivo* and clinical studies. Accumulating evidence suggests that bio-active constituents of TCM accelerate bone metabolism that is achieved via antioxidative, anti-inflammatory, and osteogenic mechanisms. Salidroside (SAL), isolated from Rhodiola, facilitates osteoblast proliferation and attenuates oxidative stress, while resveratrol ameliorates hypoxia-induced OP by suppressing the ROS/HIF axis and stimulating osteogenic differentiation (Zhang et al., 2013). Within the warming-Yang framework, Fuzi and Ganjiang exemplify metabolic activation through modulation of mitochondrial function. Modern pharmacological studies demonstrate that aconitine alkaloids and gingerols potentiate mitochondrial respiration, upregulate antioxidant enzymes, and promote osteogenesis via the HIF-1α/VEGF and AMPK signaling pathways. Thus, elucidating the mechanisms of GAWD provides a rational basis for its potential to protect osteoblasts against hypoxia-induced injury and to restore impaired bone metabolism (Deng et al., 2019; Li et al., 2021).

Building upon these principles, the present study aims to elucidate how high-altitude hypoxia induces osteoblastic injury through HIF-1 signaling dysregulation and to investigate the protective mechanisms of the GAWD. By integrating transcriptomic and functional analyses, this study focuses on osteoblast-specific responses—including oxidative stress, mitochondrial dysfunction, and apoptosis—under hypoxia, thereby providing a theoretical foundation for TCM-based prevention of high-altitude osteoporosis. Mouse calvaria-derived pre-osteoblast (MC3T3-E1) cells were selected as an *in vitro* model due to their osteogenic differentiation potential and sensitivity to environmental and mechanical stimuli (Song et al., 2022). Consequently, we established a simulated high-altitude hypoxia model to elucidate the effects of sustained hypoxia on osteoblastic function, focusing on the regulatory role of the HIF-1α–VHL–VEGF axis. Through transcriptomic analysis and functional validation, we aimed to clarify the molecular basis of hypoxia-induced osteogenic dysfunction and evaluate the protective mechanism of GAWD. These findings may provide novel insights and a theoretical foundation for developing TCM-based strategies to prevent and treat osteoporosis in high-altitude environments.

## 2 Materials and methods

### 2.1 Experimental cells and experimental drugs

The MC3T3-E1 osteoblast precursor cells used in this experiment were obtained from Wuhan Puno Sai Life Science and Technology Co., Ltd. Black Shun tablets (processed Aconitum carmichaelii Debeaux, Batch No. 2404086) and dried ginger (Zingiber officinale Roscoe, Batch No. 24071702), the two herbal components of the GAWD, were obtained from the Affiliated Hospital of Gansu University of Chinese Medicine. The preparation was carried out in accordance with the Pharmacopoeia of the People’s Republic of China (2015, Part I). Specifically, 30 g of Black Shun tablets (Fuzi) were weighed and immersed in eight times their volume of purified water for 30 min, followed by decoction for 30 min. Subsequently, 20 g of dried ginger (Ganjiang) was added, soaked for an additional 30 min, and decocted twice. The resulting decoctions were combined, filtered, and concentrated to yield a solution equivalent to 0.5 g of crude drug per milliliter. The extract was then centrifuged to remove impurities, sterilized through a 0.22 μm aqueous filter membrane, and stored at 4 °C. Before use, the decoction was diluted with the appropriate culture medium to the desired concentration.

#### 2.1.1 Main instruments and equipment

We used the following instruments with this study: a programmable positive and negative pressure cell culture incubator (Patent No. ZL202022539967.0); a flow cytometer (BriCyte E6); a real-time fluorescence quantitative PCR system (LightCycler 96); a Western blot system consisting of an electrophoresis system, a translation system, and a chemiluminescence imaging system; NovaSeq X Plus sequencing platform (Illumina). Microplate reader (Multiskan Mk3) and an inverted microscope (IX51).

#### 2.1.2 Main reagents used in the experiment

The cell culture reagents utilized in this study included α-MEM culture medium (Hyclone, United States; Catalog No. AJ30742844), fetal bovine serum (Zhengzhou Pingrui Biological Technology Co., Ltd., China; Catalog No. ZPRTN100-04), and a Penicillin-Streptomycin-Amphotericin B mixture (100×, Beijing Solabao Technology Co., Ltd., China; Catalog No. P1400). For the cell viability assay, a CCK-8 (Shenzhen Shangwei Biotechnology Co., Ltd., China; Catalog No. DCM2188) was employed. RNA extraction and sequencing were performed using TRIzol® reagent (Invitrogen, United States), an RNA purification kit (Hangzhou Guangke Ande Biological Technology Co., Ltd., China), the Illumina® Stranded mRNA Prep Ligation Kit, and the NovaSeq reagent kit (Illumina, United States), along with Biowest agarose (Biowest, Spain). Protein extraction and quantification were conducted using Efficient RIPA lysis buffer (Beijing Solabao Technology Co., Ltd., China; Catalog No. R0010), a BCA protein quantification kit (Beijing Solabao Technology Co., Ltd., China; Catalog No. PC0020), and a 5× protein loading buffer (Beijing Solabao Technology Co., Ltd., China; Catalog No. P1041). For SDS-PAGE and Western blot analysis, the following materials were utilized: a 10% color fast gel preparation kit (Saiwen Innovation, China; Catalog No. SW143-02), 5×SWE electrophoresis buffer (Wuhan Saiwei Biological Technology Co., Ltd., China; Catalog No. G2152-1L), 10×TBST buffer (Wuhan Saiwei Biological Technology Co., Ltd., China; Catalog No. G0004-1L), and a PVDF membrane (Immobilon, United States; Catalog No. IRVH00010). Additionally, a pre-stained protein marker (Saiwen Innovation, China; Catalog No. SW176-02), skimmed milk, a rapid seal blocking solution (Saiwen Innovation, China; Catalog No. SW162-02), and an ECL chemiluminescence reagent kit (Wuhan Pumeike Biotechnology Co., Ltd., China; Catalog No. PMK0448) were employed. The antibodies utilized in this study included the following: Rabbit anti-Goat IgG secondary antibody (Wuhan Pumeike Biotechnology Co., Ltd., China; Catalog No. PMK014), GAPDH internal control antibody (Jiangsu Baijia Biotechnology Co., Ltd., China; Catalog No. IDS0124), VHL antibody (Abcam, United Kingdom; Catalog No. ab270968), VEGF antibody (Jiangsu Qinko Biotechnology Co., Ltd., China; Catalog No. AF5131), HIF-1α antibody (Jiangsu Qinko Biotechnology Co., Ltd., China; Catalog No. BF8002), and HIF-1β antibody (Jiangsu Qinko Biotechnology Co., Ltd., China; Catalog No. DF3136).

#### 2.1.3 Simulation of high-altitude (hypobaric) conditions

The *in vitro* high-altitude environment was established using a self-developed adjustable positive- and negative-pressure cell culture incubator (patent No. ZL202022539967.0). This system precisely regulates internal barometric pressure and oxygen partial pressure, thereby enabling direct setting of target “altitude” parameters to reproduce the low-pressure hypoxic conditions characteristic of different high-altitude environments. Exposure to simulated 4,500-m conditions was achieved by maintaining the oxygen concentration at 3% O_2_ for 72 h, which effectively induces hypoxia-related cellular responses.

Experimental groups were maintained at equivalent altitudes of 2,500 m, 3,500 m and 4,500 m at exposure durations of 24, 48, 72, 96 and 120 h. Cells were tested for cell viability using the CCK-8 assay at each time point. Cell viability declined progressively with increasing simulated altitude and exposure duration, and the 4,500 m/72 h condition resulted in viability of about 60%. This condition was hence adopted as the best high-altitude condition for the next series of experiments.

Of note, the “4,500 m” altitude was interpreted as equal to a pressure controlled hypoxic condition corresponding to the atmospheric parameters of this elevation, rather than the actual size of a physical altitude; it was interpreted as a cellular-level model intensity but not the physiological equivalent at which the organism could be affected. The control group was cultured at local atmospheric pressure of Lanzhou (∼1,500 m). We established a series of hypoxic stress intensity by increasing the simulated altitude incrementally and found that the 4,500 m was a frequent stable threshold of osteoblastic injury.

Under drug intervention cells remained at the simulated 4,500 m altitude and administered various GAWD concentrations for 24–72 h. All treatment processes proceeded fully within the hypobaric incubator to keep environmental conditions consistent. Normal sub-culturing procedures were performed for cell handling. Finally, whether the hypoxia-mediated ‘plateau state’ is maintained after re-oxygenation was not considered here further.

#### 2.1.4 Quality control and methodological rigor

All experiments were conducted in MC3T3-E1 osteoblastic cells maintained continuously at the same passage number in order to minimize biological variation. Cell culture was carried out under sterile conditions. Before each experiment, the hypobaric incubators were calibrated to accurately mimic the high-altitudepressure (4,500 m) and oxygenpartial pressure. Before sequencing, RNA integrity was confirmed with a NanoDrop 2,000 and Agilent 5,300 Bioanalyzer (RIN >8.0). Sequencing libraries were prepared using an Illumina Stranded mRNA Prep Ligation Kit and analyzed on the NovaSeq X Plus platform. For cytological assays, such as the measurement of cell viability, ROS, Ca^2+^, MMP and apoptosis, all experiments were conducted in triplicate with identical instrument parameters. Western plots and Quantitative rt-PCR analyses were conducted following standardized protocols, and all data were validated using melting-curve and normalization controls. All of the experiments were reproduced independent of each other (n = 3) and data are presented as mean ± SD. All data were statistically significant by a one way anova followed by a Tukey’s *post hoc* test (P < 0.05).

### 2.2 Experimental methods

#### 2.2.1 Cell culture

The α-MEM culture medium was prepared by combining fetal bovine serum and 1% penicillin/streptomycin in a ratio of 90:10:1, resulting in a complete culture medium containing 10% fetal bovine serum. After thorough mixing, the medium was stored at 4 °C until needed. Prior to the experiment, the laminar flow hood was disinfected using alcohol-soaked cotton balls, and the UV light was activated for sterilization. Simultaneously, the water bath was preheated to 37 °C. The frozen MC3T3-E1 cells were promptly retrieved from the −80 °C freezer and rapidly thawed in the water bath with intermittent gentle shaking to facilitate recovery. Once only a few ice crystals remained, the cells were removed, disinfected with alcohol, and placed in the laminar flow hood. The cell suspension was transferred to a 15 mL centrifuge tube and centrifuged at 1,000 rpm for 5 min. After discarding the supernatant, 3 mL of complete culture medium was added to resuspend the cells, which were mixed gently by pipetting before being inoculated into a culture flask. The flask was gently rocked in a figure-eight or cross pattern to ensure even distribution and was subsequently incubated in a culture incubator. When the cells reached 80%–90% confluence, passaging was performed in the disinfected laminar flow hood. Prior to the experiment, PBS, complete culture medium, 0.25% EDTA-trypsin, and culture flasks were treated with 75% ethanol. After discarding the old culture medium, the cells were washed twice with 2 mL of PBS and then digested with 1 mL of trypsin. The flask was gently shaken to ensure full contact between the cells and the enzyme solution. When the cells appeared rounded and slightly shrunk under the microscope, 2 mL of complete culture medium was added to halt digestion. After gentle pipetting, a single-cell suspension was formed, and the cells were re-seeded at a ratio of 1:3 into a new T25 flask. Following gentle mixing, the flask was placed in a 37 °C, 5% CO_2_ incubator for continued culture. The status of the cells was observed daily, and the culture medium was replaced every 2–3 days. When the MC3T3-E1 cells reached 80%–90% confluence and appeared healthy, they were cryopreserved. The laminar flow hood was activated and subjected to UV disinfection, and all related reagents and materials were disinfected with 75% ethanol before being transferred into the hood. After discarding the original culture medium, the cells were washed twice with PBS and then digested with trypsin. When the cells became rounded and shrank under the microscope, complete culture medium was added to halt the digestion. Following dispersion by pipetting, the cells were transferred into a 15 mL centrifuge tube and centrifuged at 1,000 rpm for 5 min. The supernatant was discarded, and 1 mL of cryopreservation solution was added to resuspend the cells. The suspension was aliquoted into cryovials, sealed, labeled, and stored at −80 °C or in liquid nitrogen.

#### 2.2.2 CCK8 assay to detect the effect of high altitude on cell viability

MC3T3-E1 cells were seeded at a concentration of 1 × 10 5 cells/well in a 96-well plate. There were a control at 1,500 m and a series of altitude groups at 2,500 m, 3,500 m, and 4,500 m, respectively. The interventions each lasted for 24, 48, 72, 96, and 120 h, and were replicated 5 times. A blank control group with only a complete culture medium was set up as well. After the attached plate were incubated with an adjustable positive and negative pressure cell culture incubator (Patent No.: ZL 202022539967.0) for a period of time to represent the multiple altitude environments. At each time point, the 96-well plate was replaced, 100 μL of new medium was added, and 10 μL of CCK-8 reagent was added for another 2 h. The absorbance with 450 nm was recorded to analyze the viability of the cells to determine the high-altitude damage model. The RNA was divided into the TRIzol extraction method, and the quantitative amount of 1 μL of sample was measured by Nanodrop 2000. The integrity of the RNA was assessed using agarose gel electrophoresis and a Agilent 5,300. The RIN value was calculated. mRNA was fortified by Oligo (dT) magnetic beads, and fragmented into 300 bp pieces using a fragmentation buffer. cDNA was synthesized by reverse transcription utilizing the mRNA as a template andAdapter ligation step, followed by purification. After PCR amplification, the libraries were constructed. Finally, the samples were measured using Qubit 4.0, and after mixing, the sequencing of the samples was done on the NovaSeq Xplus platform through bridge PCR amplification.

#### 2.2.3 Gene expression of differential genes in MC3T3-E1 cells under high altitude conditions

The gene expression profiles of the high-altitude group and the control group were Subsequently considered all the genes with DEGs identified using the (DESeq2) software. Significant differential expression were defined as follows: FDR <0.05 and |log2 FC| ≥ 1. All the genes that met both criteria would be considered and identified as differentially expressed and would be further used for functional studies.

#### 2.2.4 GO and KEGG enrichment analysis of differential genes

GO functional enrichment and KEGG pathway analyses were done by Goatools and KOBAS. fisher’s exact test was applied and P-values were normalized by multiple testing methods (P < 0.05). The smaller the P-value, the bigger the biologically significant and important processes and pathways.

#### 2.2.5 Reactome pathway enrichment analysis

The Reactome database is a collection of diverse biological components, which includes nucleotide, protein, and complex, to build a signaling and metabolic network. The pathways involved in metabolism, signal transduction, transcriptional regulation, apoptosis, and many diseases were included. ReactomePA was employed to do the Reactome enrichment analysis to study the set of differentially expressed genes.

#### 2.2.6 Screening of the optimal concentration of Aconite-dried ginger decoction for intervention

MC3T3-E1 cells were seeded at a density of 1 × 10^5^ cells per well in a 96-well plate and divided into three treatment groups: blank, high-altitude, and Aconite-Dried Ginger Decoction (0.125, 0.25, and 0.5 mg/mL), with five replicates for each group. The blank group consisted solely of complete culture medium. After an overnight incubation, the cells were transferred to an adjustable cell culture incubator, where the high-altitude group was maintained at an altitude of 4,500 m. Various concentrations of Aconite-Dried Ginger Decoction were administered for 24, 48, and 72 h. Subsequently, 10 μL of CCK-8 solution was added to each well, and after a 2-h incubation period, the optical density (OD) at 450 nm was measured using a microplate reader to determine the optimal time and concentration.

#### 2.2.7 Optical microscopic observation of cell morphology

The culture of MC3T3-E1 cell was examined under excellent cell growth conditions with an inverted microscope. Cells on logarithmic growth phase, with defined morphology, strong capacity to proliferate, tight organization and clear border were selected for examination. Cell morphology of control, high-altitude and GAWD + high-altitude groups was recorded with optical microscope.

#### 2.2.8 Effect of Aconite-Dried Ginger Decoction on apoptosis of MC3T3-E1 cells under high altitude conditions

Experimental cells were divided into three groups: control, high-altitude, and GAWD + high-altitude. Cells were plated in 6-well plates at a plating density of 1 × 10^6^ cells/mL with a volume of 1 mL of cell suspension in each well. At 24 h plating time, cells were transferred to the adjustable cell culture incubator, where the best concentration of GAWD was added for continued cell culturing. After digestion, the cells were spun down and suspended into 100 μL of 1 × 1 Binding Buffer. 5 μL of Annexin V-FITC and 10 μL of PI were added, and the samples were maintained at room temperature in the dark from 5 to 10-min incubation. After incubation, 400 μL of 1 × binding Buffer was employed to mixed the samples. Samples were filtered with 300-mesh cell sieve and flow cytometrically assessed.

#### 2.2.9 Effect of Aconite-Dried Ginger Decoction on Ca^2+^ in MC3T3-E1 cells under high altitude conditions

Cells were divided into three categories, control, high-altitude and GAWD + high altitude. A Fleo-4 AM probe was adjusted to 1:1,000 in serum-free medium and then added to the cell suspension for 100 μL and then a 300 μL probe solution was added to the cells. Cells were incubated in the dark for 30 min. After 24 h incubation in adjustable cell culture incubator, optimal concentration of GAWD was added for further culture. The cells were digested, centrifuged, resuspended in 5 mL of PBS, and centrifuged at 300 *g* for 5 min to remove unbound probe. The cells were res suspend in 300 μL of PBS for detection by flow cytometry.

#### 2.2.10 Flow cytometry to detect ROS levels in cells from each group

Cells were divided into control, high-altitude, and GAWD + high-altitude groups. ROS levels in each groups of cell was was quantified using a ROS assay kit. Cells plated in 6 witth plate and cultured for 24 h. They were then transferred to an adjustable cell culture incubator. Then the optimal concentration of GAWD was added in order to maintain the culture condition. DCFH-DA was diluted in serum-free culture medium in a 1:1,000 ratio and 100 μL of the cell suspension was mixed with 300 μL of probe. Then, Inc. incubation for 30 min in the dark. Upon centrifugation, cells were suspended in phosphate buffered saline (PBS) and unbound probes removed after washing. AfterwardsFlow cytometry performed for quantitating the cells ROS level. The data was analysed using MRFLOW and FlowJo™ vX Software.

#### 2.2.11 Effect of Aconite-Dried Ginger Decoction on mitochondrial membrane potential in MC3T3-E1 cells under high altitude conditions

The cells were categorized into control, high-altitude, and GAWD + high-altitude groups and subsequently tested using the JC-10 probe. The JC-10 storage solution was diluted at a ratio of 1:1,000 with Hanks’ Balanced Salt Solution (HBSS), resulting in a working concentration of 30 μM. Cells were seeded in 6-well plates and cultured for 24 h before being transferred to an adjustable cell culture incubator. The optimal concentration of GAWD was then added to continue the culture process. After digestion, 5 mL of PBS was added, and 100 μL of the cell suspension was mixed with 300 μL of the JC-10 probe, followed by a 30-min incubation in the dark. After centrifugation, the cells were resuspended in PBS, and any unbound probe was washed away. Flow cytometry was subsequently employed to assess the mitochondrial membrane potential.

#### 2.2.12 Effect of Aconite-Dried Ginger Decoction on cell cycle in MC3T3-E1 cells under high altitude conditions

The cells were divided into control, high-altitude, and GAWD + high-altitude groups to analyze cell cycle changes using propidium iodide (PI) staining. Cells were seeded in 6-well plates and cultured for 24 h before being placed in an adjustable cell culture incubator. The optimal concentration of GAWD was then added to continue the culture. Following digestion, cells were centrifuged, and the supernatant was discarded. One milliliter of pre-cooled PBS was added to resuspend the cells, and the suspension was centrifuged again. One hundred microliters of cell suspension was taken per tube, and five hundred microliters of DNA staining solution and 10 μL of permeabilization solution were added. The mixture was vortexed and incubated in the dark for 30 min. Flow cytometry was conducted at the lowest sample loading speed to analyze the cell cycle.

#### 2.2.13 RT-qPCR to measure HIF-1α, HIF-1β, VHL, and VEGF expression levels in cells from each group

Two hundred microliters (200 μL) of Trizol Reagent were added to a T25 culture flask, and the cells were scraped off and transferred to a new tube. After repeated pipetting, the samples were incubated at room temperature for 5 min. The mixture was then centrifuged at 12,000 rpm at 4 °C for 5 min. The supernatant was carefully transferred and mixed with chloroform in a 5:1 ratio. Following thorough mixing, the solution was left at room temperature for an additional 5 min and then centrifuged again at 12,000 rpm at 4 °C for 15 min. The RNA-containing supernatant was collected and combined with an equal volume of isopropanol, then incubated at room temperature for 10 min. The samples were centrifuged at 12,000 rpm at 4 °C for 10 min, and the supernatant was discarded. The RNA pellet was washed with pre-cooled 80% ethanol and centrifuged at 7,500 rpm at 4 °C for 5 min. After discarding the supernatant, the RNA pellet was dried for 10 min. The RNA was then dissolved in RNase-free water, and its concentration and purity were measured to ensure that the A260/A280 ratio was between 1.8 and 2.1, thereby confirming the accuracy of subsequent experimental results. Genomic DNA was removed, and reverse transcription was conducted for further analysis.

##### 2.2.13.1 Removal of gDNA

The reaction solution was prepared on ice according to the Tables 1, 2 below for the gDNA removal reaction.

**TABLE 1:** Reverse Transcription reaction system (prepared on ice).

Reagent	20 μL reaction system
gDNA clean reaction mix Ver.2	2 μL
Amount of RNA template	<1 μg (The dosage was calculated according to the concentration)
RNase-free water	It was supplemented to 16 μL

After the pipettes were mixed gently, the reaction was allowed to proceed for 2 min at 42 °C. (2) Reverse transcription reaction.

**TABLE 2:** Reverse Transcription reaction system (prepared on ice).

Name of component	20 μL reaction system
Step one reaction solution	16 μL
5X Evo M-MLV RT reaction mix Ver.2	4 μL

The reaction was carried out on the PCR, reaction instrument under the following conditions: 37 °C for 15 min, 85 °C for 5 s. The synthesized cDNA, was stored in a −20 °C refrigerator for subsequent experiments.

The reverse transcription reaction system was prepared on ice according to the table below, and the reverse transcription reaction was carried out on a PCR instrument.

##### 2.2.13.2 Real-time fluorescent quantitative PCR

1. The primers HIF-1α, Hif-1α, VHL, VEGF and GAPDH were designed and synthesized by Beijing Qingke Biotechnology Co., LTD. The sequences are shown in Table 3.
2. The following reaction system is configured, as shown in Table 4.
3. Reaction conditions, see Table 5.

**TABLE 3:** Primer sequence design.

Gene	Primer sequence	Length
HIF-1α	Forward:TGATGTGGATAGCGATATGGTCA Reverse:GAAAATGGATTCTTTGCCTCTG	23 22
VEGF	Forward:GCAGACCAAAGAAAGACAGAACA Reverse:ACAGTGAACGCTCCAGGATTTA	23 22
HIF-1β	Forward:AAGGTCAGGTGCTGTCCGTC Reverse:CTGGCTAGAGTTCTTCACATTGGT	20 24
VHL	Forward:TGAAGGCACCGCTCTTTCA Reverse:GCTACCGAGGTCATCTTTGGC	19 21
GAPDH	Forward:TGTTTCCTCGTCCCGTAG Reverse:CAATCTCCACTTTGCCACT	20 21

**TABLE 4:** Reverse Transcription reaction system (prepared on ice).

Reagent	20 μL system	Final concentration
2X SYBR green Pro Taq HS Premix	10 μL	1×
Upstream Primers,10 μM	0.4 μL	0.2 μM
Downstream Primers,10 μM	0.4 μL	0.2 μM
Template cDNA	2 μL	-
RNase-free water	Up to 20 μL	-

**TABLE 5:** Reaction conditions for RT-qPCR.

Procedure of reaction	Temperature	Time	Number of cycles
1	95 °C	30 s	1
2	95 °C	5 s	40 cycles
	60 °C	30 s	
3	95 °C	15 s	1
	60 °C	1 min	
	95 °C	1 s	

Following the reaction, the RT-PCR amplification curve and the dissolution curve were verified to ensure the reliability of the obtained data. The 2^−ΔΔCT^ method was employed for the relative quantification of the target gene.

#### 2.2.14 Western blot analysis

Cells were rinsed with 3 mL of ice-cold PBS (0.01 M, pH 7.2–7.3) per flask. Subsequently, the cells were lysed on ice using 400 μL of lysis buffer containing PMSF for 30 min, with occasional scraping to dislodge any remaining cells. The lysates were then transferred to 1.5 mL microcentrifuge tubes and centrifuged at 12,000 rpm for 10 min at 4 °C. The supernatant was collected, aliquoted into fresh tubes, and stored at −20 °C until further use. Protein concentration was determined using the BCA assay. Briefly, BCA Reagent and Cu Reagent were mixed in a 50:1 ratio to prepare 10 mL of working solution. A 0.5 mg/mL BSA stock solution was prepared by diluting 10 μL of BSA standard in 90 μL of PBS. Standards (0, 2, 4, 6, 8, 12, 16, and 20 μL) were added to a 96-well plate, and PBS was added to each well to achieve a total volume of 20 μL. Samples were diluted as necessary, with 20 μL of each sample added to the corresponding wells. Each well then received 200 μL of the BCA working solution. After a 30-min incubation at 37 °C, absorbance was measured at 562 nm, and protein concentrations were calculated from the standard curve. For sample preparation, 25 μL of 5× loading buffer was mixed with 100 μL of each protein sample in 200 μL tubes, and the samples were denatured at 100 °C for 5 min in a water bath. Prepared samples were subsequently stored at −20 °C or −80 °C if not used immediately. Glass plates and gel casting equipment were thoroughly cleaned, dried, and assembled with the thinner plate positioned on the outside. A 10% resolving gel was poured first, ensuring no air bubbles formed, and allowed to polymerize for 30 min. A 5% stacking gel was poured on top, and a comb was inserted; this assembly was allowed to set for an additional 30 min. For electrophoresis, thawed denatured samples were vortexed, briefly centrifuged, and 5 μL were loaded per lane. The gel was run at a constant voltage of 80 V (approximately 400 mA) for 30 min until the samples entered the resolving gel, followed by an increase to 120 V for approximately 100 min. Proteins were transferred to PVDF membranes that had been pre-activated in methanol using the wet transfer method. Transfer sandwiches were assembled in the following order: sponge, filter paper, gel, PVDF membrane, filter paper, sponge. The transfer was conducted at a constant current of 300 mA for 90 min. Membranes were blocked for 2 h at room temperature and then incubated overnight at 4 °C with the primary antibody. After three 5-min washes in TBST, membranes were incubated with the secondary antibody at room temperature for 90 min. Following three additional washes with TBST, ECL substrate was applied evenly, and chemiluminescence was detected. Band intensities were quantified using ImageJ software to determine relative protein expression.

#### 2.2.15 Statistical analysis

All data were analyzed using SPSS version 25.0 (IBM Corp., Armonk, NY, United States). Comparisons between groups weredone by one-way analysis of variance (ANOVA) for variables that had a normal distribution and homogeneous variances. When the data were non-normally distributed or had unequal variances, the Wilcoxon–Mann–Whitney rank-sum test was used. Graphs were constructed using GraphPad Prism version 8 (GraphPad Software, La Jolla, CA, United States). A two-tailed P value less than 0.05 was deemed to be statistically significant.

## 3 Results

### 3.1 Cell viability under different altitudes and exposure durations

**FIGURE 1:**
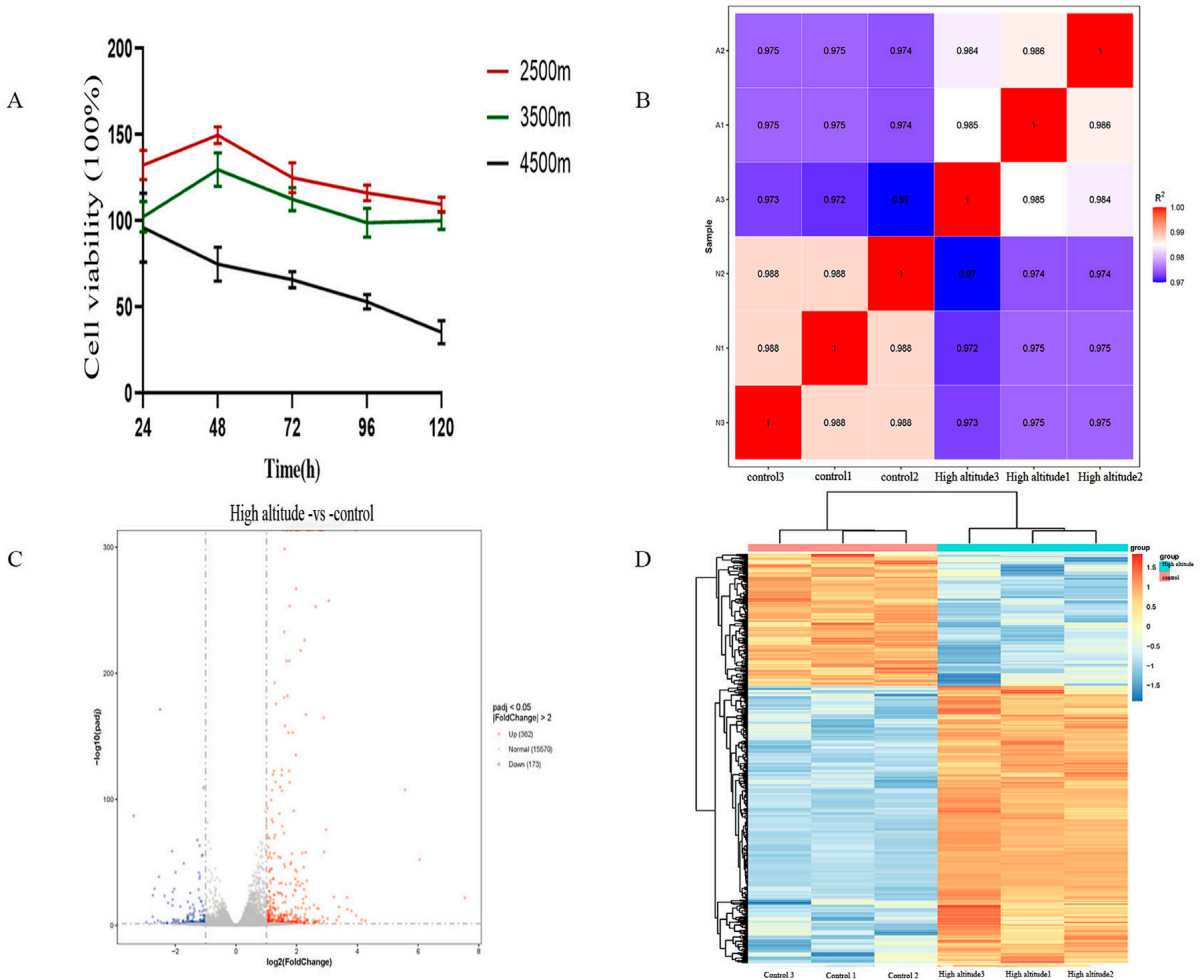
Panel **(A)** shows the biological activity of MC3T3-E1 cells in response to different accelerated environments at high altitude with different exposure delay. Panel **(B)** is the heatmap of sample-to-sample Pearson correlation coefficients between the FPKM values. Panel **(C)** indicates the volcano plot of differentially expressed genes whose fold change is greater than 2 and p-value is less than 0.05. Finally, Panel **(D)** is the hierarchical clustering heatmap presenting gene expression patterns across the samples.

The viability of MC3T3-E1 osteoblasts was tested after 72-h exposure to simulated altitudinal elevations of 2,500 m, 3,500 m, and 4,500 m (Lanshou, 1,500 m, acting as a control condition). A higher viability was measured for 2,500 m and 3,500 m groups (P < 0.05), relative to the control faction. In sharp contrast, the 4,500 m group presented a substantial reduction in viability (P < 0.05). Thus, exposure to 4,500 m imposed cellular damage and inhibited proliferation. In addition, viability was reduced progressively with increased inflammation time. Based on these findings, the 4,500 m exposure for 72 h—during which viability decreased to approximately 60%—was selected for all subsequent experiments (Figure 1A).

### 3.2 Transcriptomic analysis of MC3T3-E1 cells under high-altitude hypoxia

#### 3.2.1 Sample correlation

Overall Gene expression has inherent biological variation that is detectable between individual samples. We analyzed the reliability of the biological replicates through the Pearson correlation coefficient (R), with a higher R^2^ value representing stronger reproducibility (value within 1). Probability density distribution plots revealed highly concordant expression profiles among groups, with no samples identified as outliers. Transcriptome-wide analysis found that the pairwise gene expression correlation coefficient between the control and high-altitude groups was greater than 0.97 suggesting a high level of similarity. The sample-to-sample FPKM correlations visualized through the heatmap are shown in Figure 1B.

#### 3.2.2 Differentially expressed genes

The DESeq2 package was used for differential gene expression analysis between the control and high-altitude groups. Genes were considers significantly differentially expressed if the absolute fold changes were greater than 2 and with p-value less than 0.05. A total of 535 genes fitted this profile, out of which 362 upregulators and 173 Down-regulated genes seen in high-altitude group. Volcano plot (Figure 1C) and heatmap (Figure 1D) clearly show the distribution and clustering of the differentially expressed genes.

The figures of our results are shown in Figure 1. Panel A shows the biological activity of MC3T3-E1 cells in response to different accelerated environments at high altitude with different exposure delay. Panel B is the heatmap of sample-to-sample Pearson correlation coefficients between the FPKM values. Panel C indicates the volcano plot of differentially expressed genes whose fold change is greater than 2 and p-value is less than 0.05. Finally, Panel D is the hierarchical clustering heatmap presenting gene expression patterns across the samples.

#### 3.2.3 GO functional enrichment analysis

**FIGURE 2:**
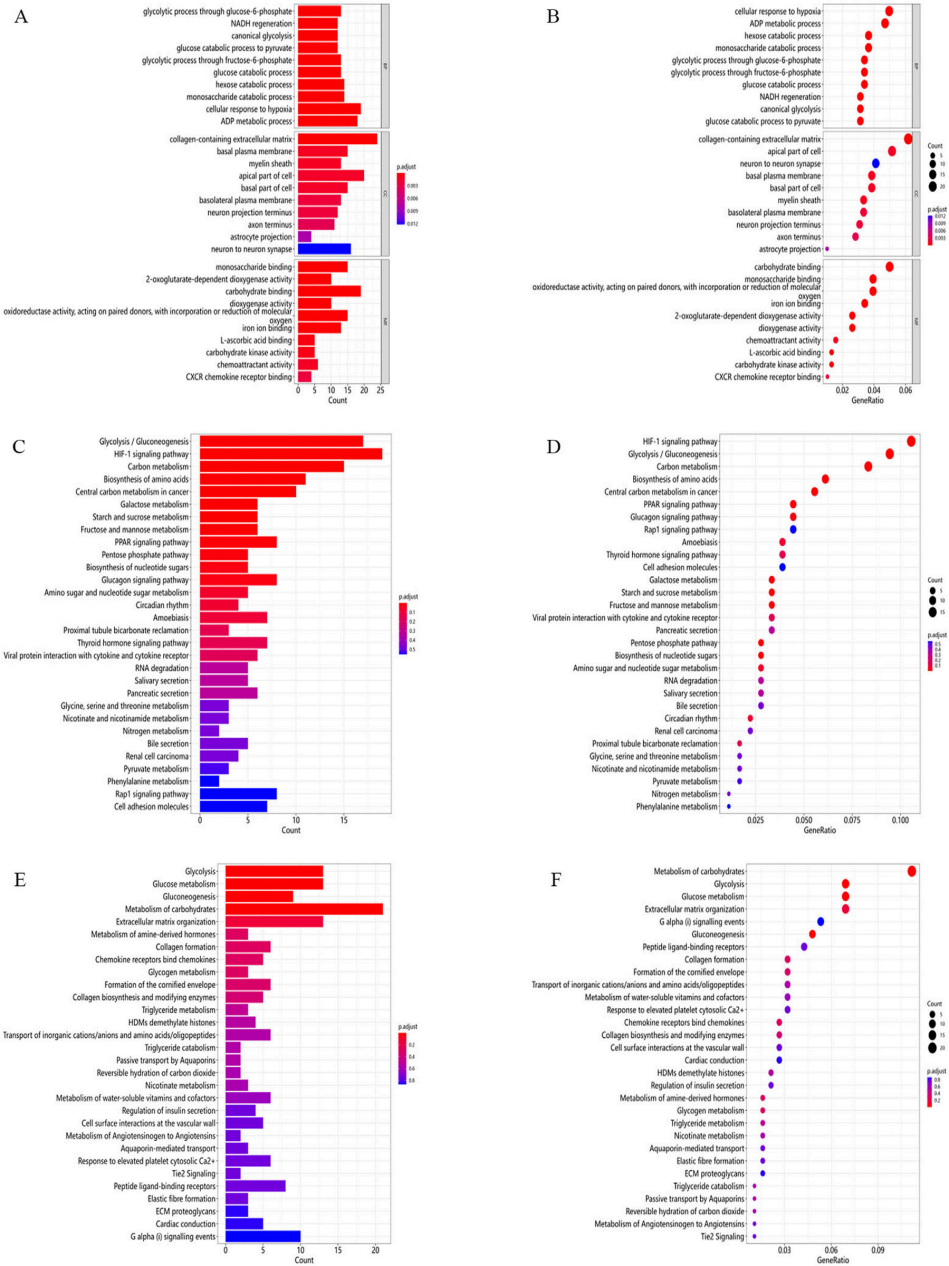
Differentially expressed genes enrichment analysed as: **(A)** Bar diagram for GO enrichment; **(B)** Bubble diagrams for GO enrichment; **(C)** Bar diagram for Kyoto Encyclopedia of Genes and Genomics (KEGG) enrichment; **(D)** Bubble diagram for KEGG enrichment; **(E)** Bar diagram for Reactome enrichment; and **(F)** Bubble diagram for Reactome enrichment.

Results of enrichment analyses were visualized using the ClusterProfessional package that used the combination of the bar plot and the bubble chart. GO analysis detected that genes differentially expressed were enriched in glycolytic pathway, NADH regeneration, glucose metabolism, monosaccharide metabolism, hypoxic response, and ADP metabolism. The adjusted P-value (padj)/padj-values showed that the significance of the genes were enriched, the lower padj-value, the more statistically significance. The detailed results of enrichment analyses were shown in the GO bar plot (Figure 2A) and the bubble chart (Figure 2B).

#### 3.2.4 KEGG pathway enrichment analysis

The KEGG pathway analysis of the differentially expressed genes identified a total of 252 significantly enriched signaling pathways. Among these, the most prominent pathways included glycolysis/gluconeogenesis, HIF-1 signaling, carbon metabolism, amino acid biosynthesis, galactose metabolism, PPAR signaling, the pentose phosphate pathway, glucagon signaling, amino sugar and nucleotide sugar metabolism, thyroid hormone signaling, RNA degradation, pyruvate metabolism, as well as the Rap1, Ras, PI3K-Akt, AMPK, MAPK, IL-17, estrogen, Apelin, TNF, Wnt, VEGF, FoxO, mTOR, and JAK-STAT pathways. Detailed enrichment results are illustrated in the KEGG bar plot (Figure 2C) and bubble chart (Figure 2D).

#### 3.2.5 Reactome pathway enrichment analysis

Differentially expressed genes were analyzed for pathway enrichment utilizing the ReactomePA package. The significant pathways identified include glycolysis, glucose metabolism, gluconeogenesis, carbohydrate metabolism, collagen formation, regulation of insulin secretion, triglyceride catabolism, cell-surface interactions at the vascular wall, elastic fiber formation, extracellular matrix (ECM) proteoglycan assembly, and Gα(i) signaling events. Detailed enrichment results are illustrated in the Reactome bar plot (Figure 2E) and bubble chart (Figure 2F).

### 3.3 CCK-8 assay of GAWD-Treated MC3T3-E1 cell viability

**FIGURE 3:**
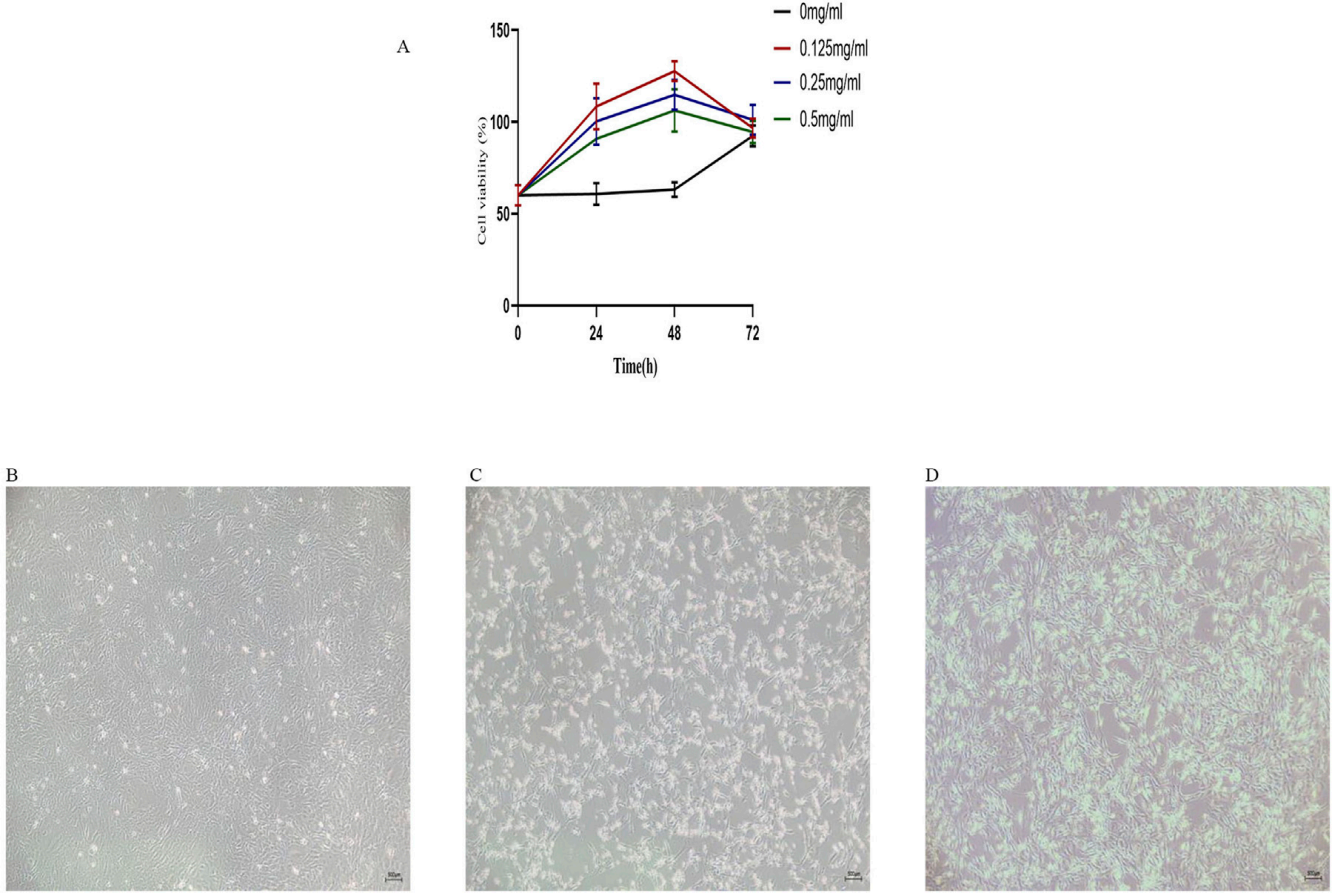
**(A)** Survivability of MC3T3-E1 cells in high-altitude hypoxia after different doses of GAWD (0, 0.125, 0.25, 0.5 mg/mL) after 24, 48, and 72 h. Representative light microscopy images (40×) of the morphology of MC3T3-E1 cells for different groups as: **(B)** Control; **(C)** High-flight hypoxia; and **(D)** GAWD combined with high-flight hypoxia.

MC3T3-E1 cells cultured for 72 h at apparent simulated altitude of 4,500 m were divided into a control group (Com, complete medium, 0 mg/mL GAWD) and treatment groups treated with GAWD at 0.125, 0.25, or 0.5 mg/mL for 24, 48, or 72 h. The best cell viability was obtained with 0.125 mg/mL concentration at 48 h, but the cell viability decreased in all treatment groups after 48 h. The cell viability did not decrease in the Com group, although it increased after 48 h, which indicates that cell injury by high-altitude stress occurred within 48 h and cell recovery after 48 h. According to this result, the GAWD treatment parameters of 0.125 mg/mL in a 48 h period were applied for further experiments (Figure 3A).

### 3.4 Light-microscopic examination of MC3T3-E1 cell morphology

At ≤×40 magnification, the control cells adhered thoroughly to the culture plate, presenting heterogeneous morphologies, with triangular, polygonal, and elongated spindle shapes. Cells preserved the intact shapes, well-organize into a ‘cobblestones’ monolayer, and proliferate extensively. In contrast, the high-altitudes hypoxic cells exhibited a loose attachment between cells, larger intercellular spaces, disorganized pattern, poor adherence, increased necrotic necrosis, and distorted shapes. Once treated by GAWD, cell–cell junctions became improved, intercellular space decreased, the cell density was increased, and the cell structures generally regained their integrity (Figures 3B–D).

### 3.5 Effect of GAWD on MC3T3-E1 cell apoptosis under high-altitude hypoxia

**FIGURE 4:**
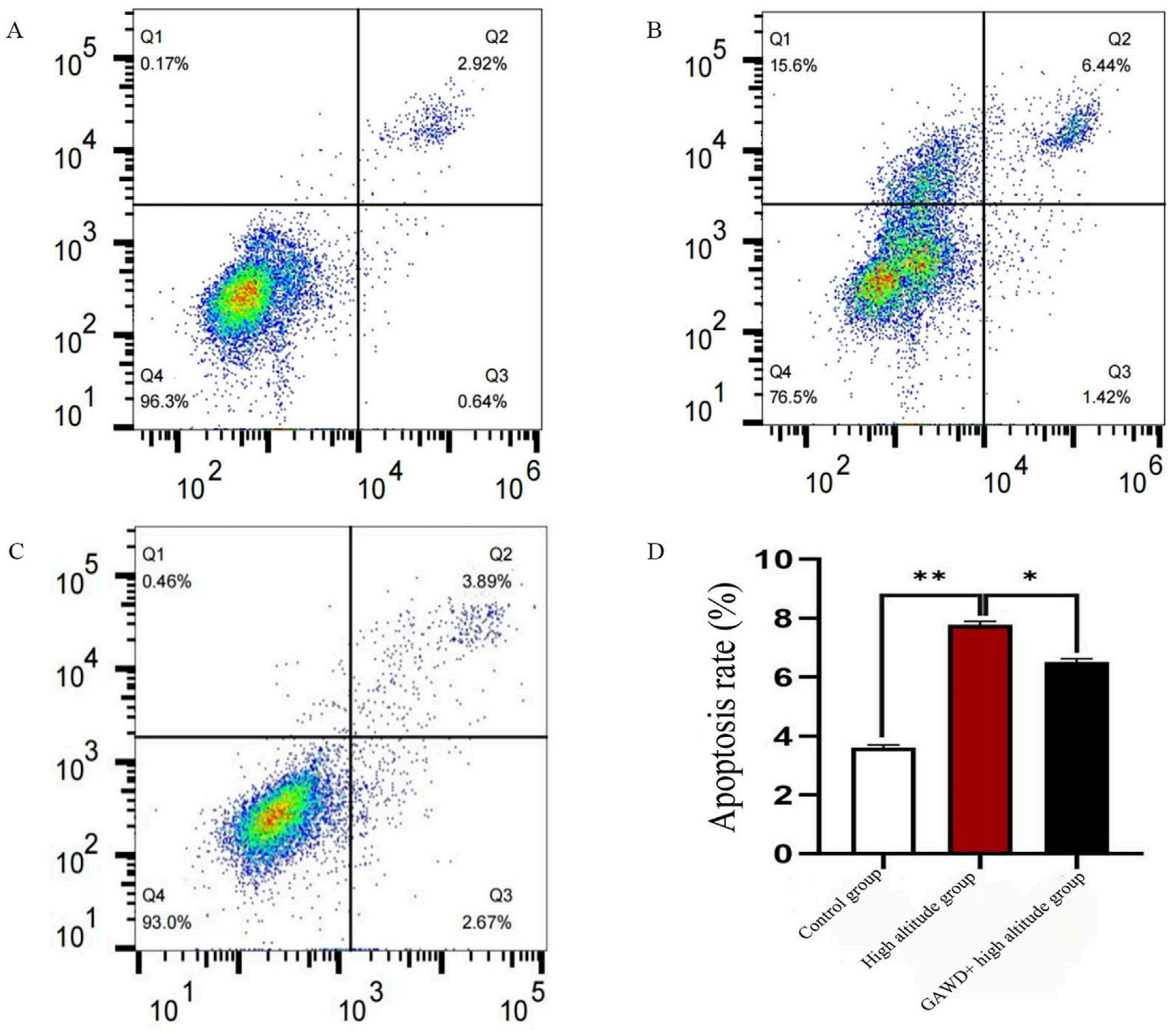
The cytometric analysis of apoptosis of MC3T3-E1 cells. **(A)** Control, **(B)** High-altitude hypoxia and **(C)**, GAWD combo with high-altitude hypoxia. The corresponding bar graph in Panel (D) shows the overall rates of apoptosis. The level of statistical significance was considered as *P < 0.01 relative to the Control and P < 0.05 relative to High-altitude.

The percentage of apoptotic cells and necrotic cells were determined by flow cytometry. Cells were categorized into four quadrants: Q1 (necrotic cells), Q2 (late apoptotic cells), Q3 (early apoptotic cells), and Q4 (viable cells), based on their fluorescence intensity. As shown in Figure 4, control cells prompted 3.56% total apoptosis. A high-altitude hypoxia was found to elevate both the rate of apoptosis (7.86%) and necrosis (15.6%) of cells. GAWD treatment under a hypoxic condition decreased the rate of apoptosis to 6.56% (P < 0.05). The results predict that exposures to a high-altitude environment promote apoptosis and necrotic proliferation of MC3T3-E1 cells, and that treatment with GAWD could partially decrease the apoptotic process of cells.

Flow lymphocyte cytometry Figures. Figure 4 shows the cytometric analysis of apoptosis of MC3T3-E1 cells. The panels 1–3 show representative dotplots of (A) Control, (B) High-altitude hypoxia and (C), GAWDcombo with high-altitude hypoxia. The corresponding bar graph in Panel D shows the overall rates of apoptosis. The level of statistical significance was considered as *P < 0.01 relative to the Control and P < 0.05 relative to High-altitude.

### 3.6 Effect of GAWD on intracellular Ca^2+^ levels in MC3T3-E1 cells under high-altitude hypoxia

**FIGURE 5:**
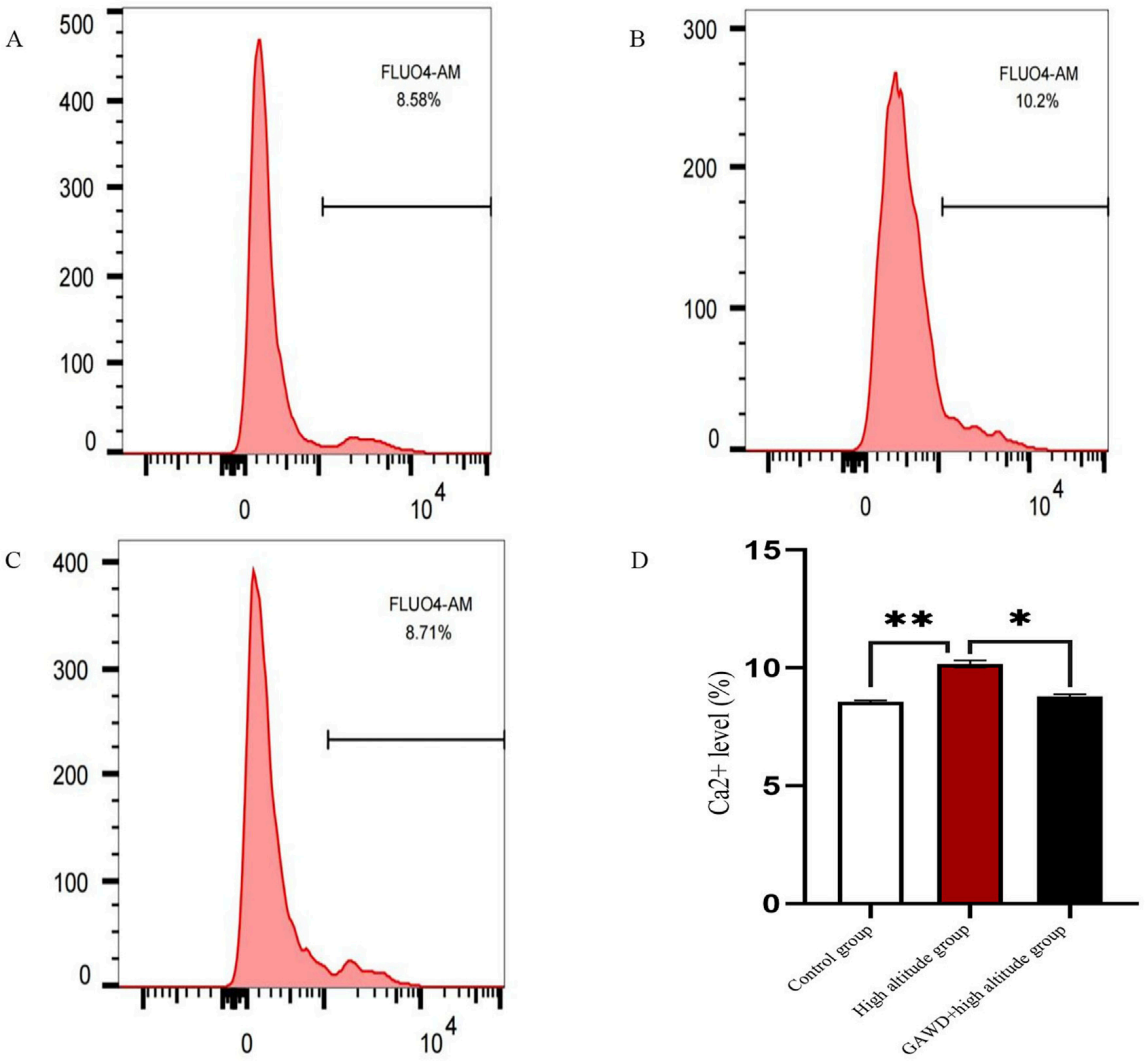
Quantification of intracellular Ca^2+^ using Fluo-4 AM flow cytometry Panels 1, 2, and 3 show representative fluorescence histograms of **(A)** Control; **(B)** High altitude hypoxia; and **(C)** GAWD plus high altitude hypoxia. Panel **(D)** shows a bar plot expression for average Ca^2+^ positive cell percentages. *P < 0.01 When compared to Control and *P < 0.05 When compared to High altitude.

MC3T3-E1 were loaded with the Fluo-4 AM calcium probe, which fluoresce green when it bound Ca^2+^ We quantified intracellular levels of Ca^2+^ using flow cytometry. The ratio of Fluo-4-positive cells was 8.58% in the control group and 10.2% (P < 0.05) in high-altitude hypoxia, indicating that Ca^2+^ overload occurred in the hypoxic conditions. GAWD treatment during hypoxic conditions decreased intracellular Ca^2+^ levels compared to high-altitude untreated group. Therefore, GAWD appears capable of effectively attenuating hypoxia-induced Ca^2+^ invasion. The findings indicate that high-altitude hypoxia causes activation of Ca^2+^ channels and Leads to Ca^2+^ overload thus worsening cellular damage (Figure 5).

### 3.7 Measurement of intracellular ROS levels by flow cytometry

**FIGURE 6:**
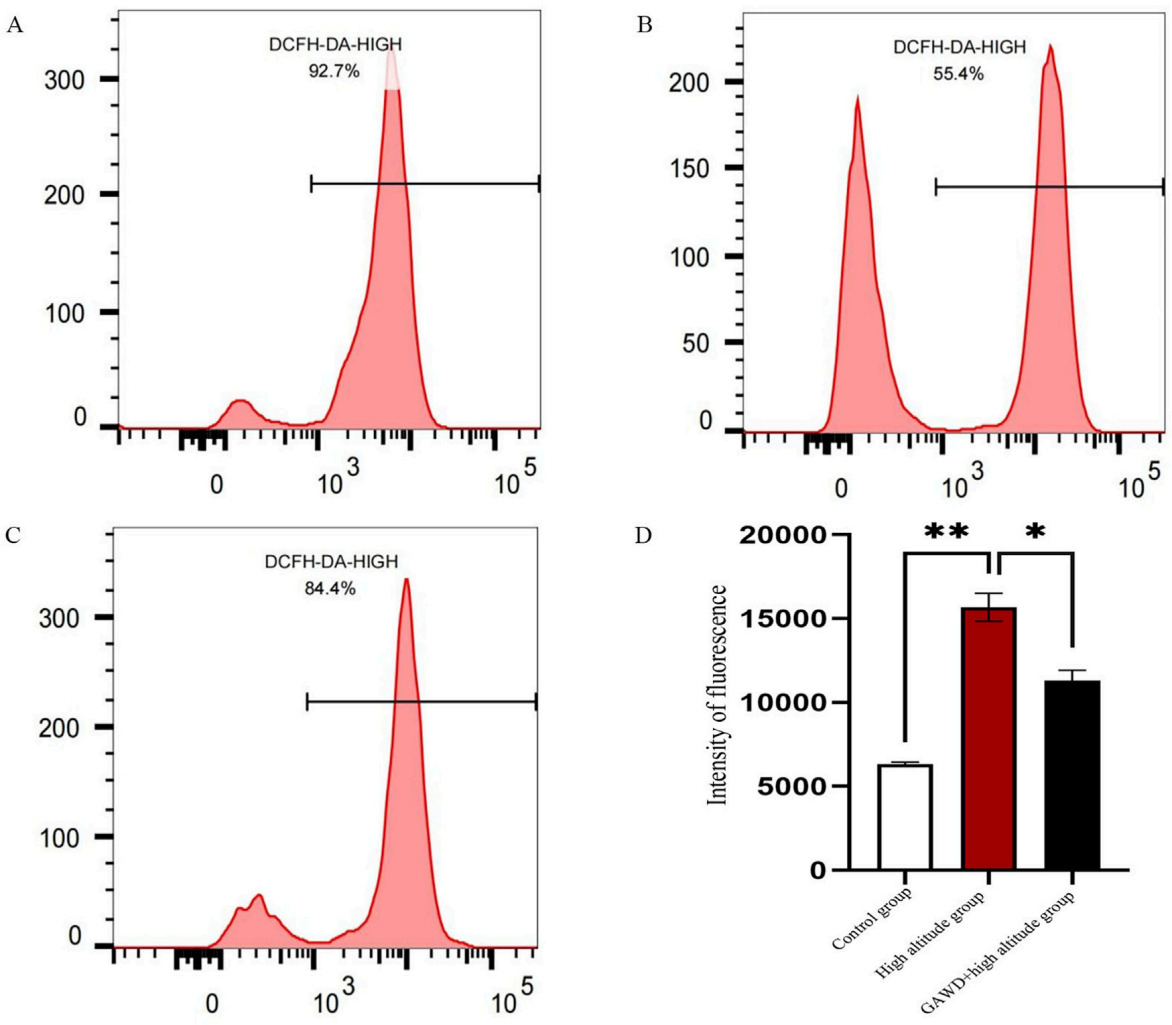
Measurement of intracellular ROS is done through flow cytometry of DCFH-DA. Representative ROS fluorescence histograms as shown in the panels are for the following experimental conditions: **(A)** control; **(B)** high-altitude hypoxia; **(C)** GAWD associated with high-altitude hypoxia. Panel **(D)** shows bar graph of the mean fluorescence intensity. *P < 0.01, versus Control; *P < 0.05, versus High-altitude.

Cells were irradiated with the DCFH-DA probe and then analyzed by flow cytometry to determine the level of ROS; Exposure to induced high-altitude hypoxia increased the ROS level in MC3T3-E1 cells dramatically, while it was not also increased in the control group (P < 0.05). Treatment of the MC3T3-E1 cells in the presence of hypoxic GAWD had a significant inhibitory effect on the intracellular ROS accumulation, indicating that GAWD is effective for treating altitude-induced oxidative stress. These results proved that the hypoxia induced at high altitude significantly increases ROS levels, resulting in cellular injury and necrosis. The high-altitude group had a survival rate of 55.4%, although it showed a higher fluorescence intensity than that of the control group (Figure 6).

### 3.8 Mitochondrial membrane potential alterations across treatment groups

**FIGURE 7:**
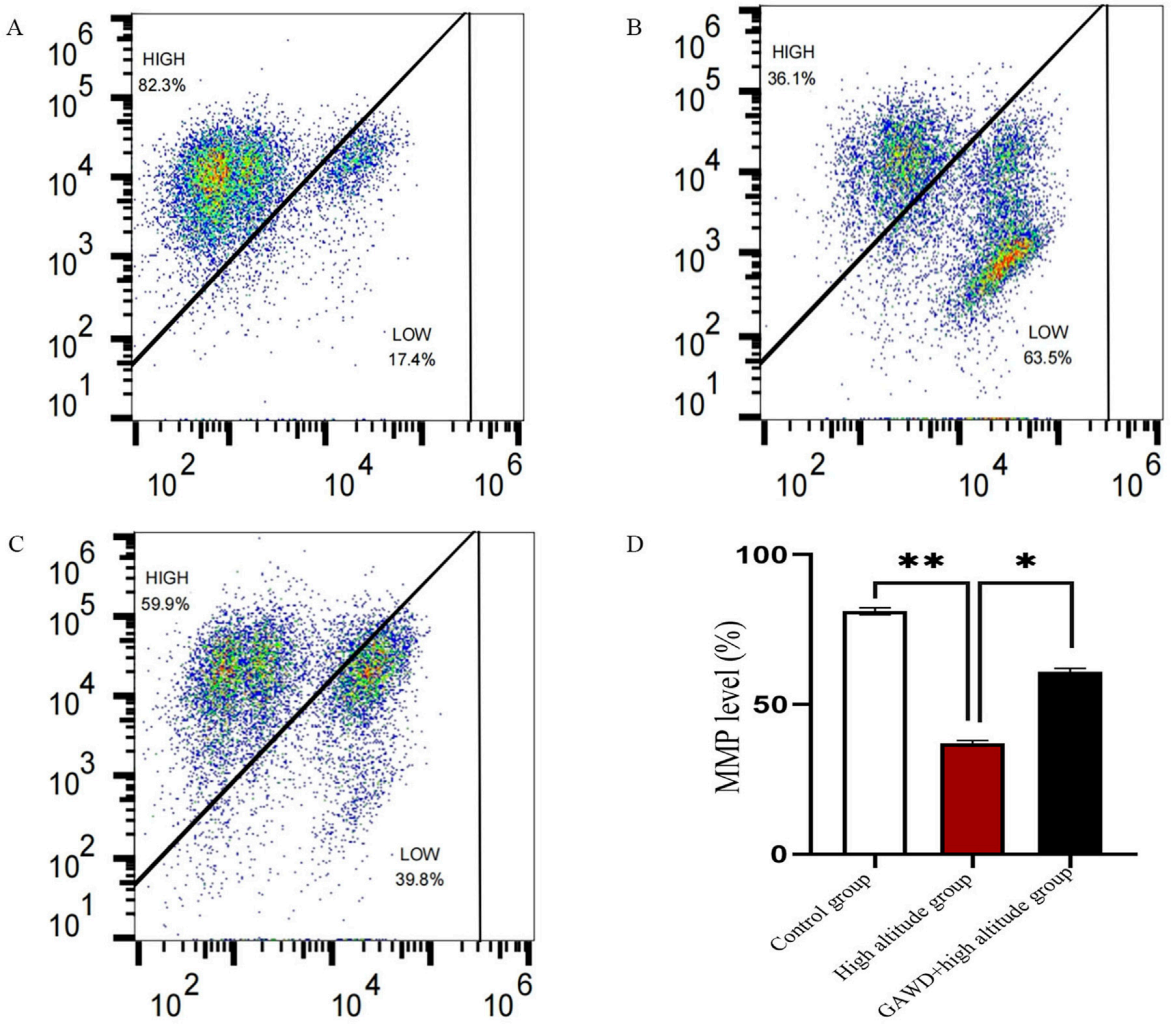
Displays the MMP calculation via JC-10 and flow cytometry. Panels 1-3 are representative red/green fluorescence plots under the following conditions: **(A)** Control; **(B)** High-altitude hypoxia; **(C)** GAWD and high-altitude hypoxia. Panel **(D)** shows the bar percentage of cells with preserved M MPs Statistical significance was determined as *P < 0.01 compared to Control and *P < 0.05 compared to High-altitude.

MMP of MC3T3-E1 cells was measured using flow cytometry with the JC-10 probe. Normal cells have green-red fluorescent MMP, while dissipated cells are green in fluorescence. Under high altitude hypoxia, the percentage of cells with intact MMP declined from 82.3% in the control to 36.1% (P < 0.05), for which the apoptotic fraction increased. GAWD treatment resulted in dramatically improved MMP in hypoxic conditions. This data suggest that the GAWD treatment is capable of reversing the hypoxia-induced apoptotic signalling. The results indicate that high altitude exposes the mitochondria-mediated apoptosis, which can be suppressed by the GAWD administration (Figure 7).

### 3.9 Cell cycle distribution across treatment groups

**FIGURE 8:**
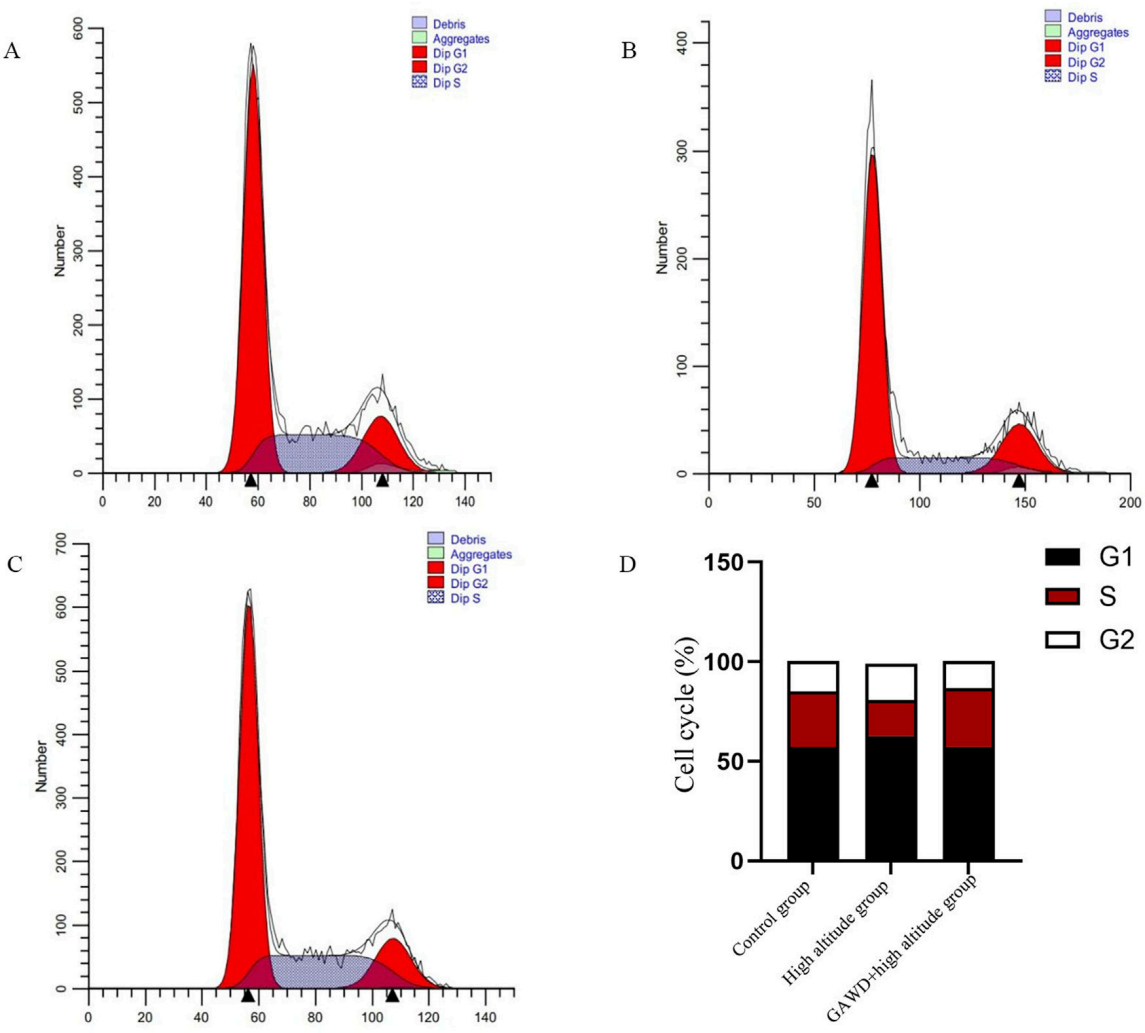
Cell cycle distribution of MC3T3-E1 cells examined under multiple variants. Flow cytometry histograms yielded representative figures for the following conditions: **(A)** Control; **(B)** High-altitude hypoxia; **(C)** Gawd in combination with high-altitude hypoxia. Panel **(D)** shows percent of cells in each cell cycle phase. All 3 were statistically significant with *P < 0.01 when compared with Control and P < 0.05 when compared with High-altitude.

Flow cytometry was employed to determine the progression of cells through cell cycle. Hypoxic conditions (high altitude) induced an arrest in the G_1_-phase, visible as an increased presence of the G_1_ population and a corresponding diminution of S-phase cells (P < 0.05). After being treated with GAWD under hypoxic conditions, we observed an increase in the percentage of S-phase cells, and a concomitant decrease in the G_1_ fraction, thus reversing the G_1_ arrest induced by high altitude (P < 0.05 and Figure 8).

### 3.10 Expression levels of HIF-1α, HIF-1β, VHL, and VEGF in MC3T3-E1 cells

**FIGURE 9:**
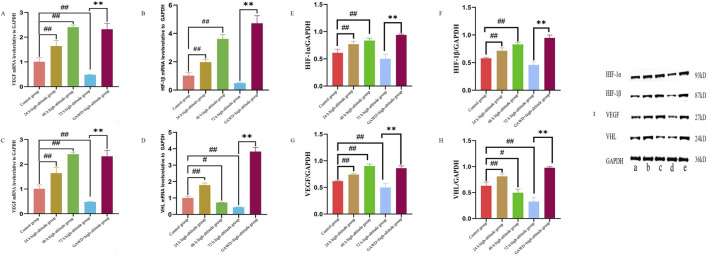
**(A–D)** The mRNAExpressions of HIF-1α, HIF-1β, VHL, and VEGF relative levels in MC3T3-E1 were measured after high-altitude condition and GAWD treatment using RT-qPCR. *#P < 0.05, ##P < 0.01 compared with Control; and P < 0.01 comparing with High-altitude 72 h. **(E–H)**. Quantification of the Western blot of HIF-1α, HIF-1β, VEifi, and VEfi proteins. *#P < 0.05, ##P < 0.01 compared with Control; and *P < 0.01 comparing with High-altitude 72 h. **(I)** Representative Western blot images of the expression of HIF-1α, HIF-1β, VHL, and VEGFi proteins in MC3T3-E1 under various conditions: **(A)** Control; **(B)** High-altitude 24 h; **(C)** High-altitude 48 h; **(D)** High-altitude 72 h; **(E)** High-altitude combined with GAWD.

Relative mRNA levels of HIF-1α, HIF-1β, VHL, and VEGF were measured by RT-qPCR. The data are presented in Table 6. The expression of HIF-1α, HIF-1β, and VEGF was significantly upregulated in the hypoxia group treated with GAWD at 24 h and 48 h, comparison to the untreated hypoxia group (P < 0.01). The expression at 72 h was drastically reduced (P < 0.01). VHL mRNA levels were increased at 24 h but significantly decreased at 48 h and 72 h (P < 0.05). At 72 h after hypoxia treatment with GAWD, HIF-1α, HIF-1β, VEGF, and VHL mRNA levels were finally restored to higher levels compared tohypoxia untreated value (P < 0.01) (Figures 9 A-D).

**TABLE 6:** Effect of high-altitude hypoxia on mRNA expression levels of HIF-1α, HIF-1β, VHL, and VEGF in MC3T3-E1 Cells ( $\bar{x}\pm s$ ).

Group	HIF-1α	HIF-1β	VHL	VEGF
Control	1.003 ± 0.093	1.015 ± 0.216	1.003 ± 0.102	1.008 ± 0.157
High altitude 24 h	5.266 ± 0.674^##^	1.973 ± 0.204^##^	1.784 ± 0.129^##^	1.643 ± 0.224^##^
High altitude 48 h	10.712 ± 0.602^##^	3.613 ± 0.322^##^	0.722 ± 0.023^#^	2.407 ± 0.085^##^
High altitude 72 h	0.418 ± 0.039	0.482 ± 0.047	0.431 ± 0.024^##^	0.476 ± 0.025^##^
GAWD + High altitude 72 h	11.454 ± 0.892^**^	4.716 ± 0.0.534^**^	3.825 ± 0.257**	2.322 ± 0.240^**^

In comparison to the control group, the results indicate a statistically significant difference, with # denoting P < 0.05 and ## indicating P < 0.01. Furthermore, when compared to the high altitude 72-h group, ** signifies a P < 0.01.

### 3.11 Protein expression analysis

Relative expression of HIF-1α, HIF-1β, VHL, and VEGF by Western blotting of MC3T3-E1 cells. The data are presented in Table 7. Western blotting of MC3T3-E1 cells was similar to the convention. Both 24 hours and 48 hours of hypoxic hypoxia increased the expressions of HIF-1α, HIF-1 β, and VEGF proteins significantly (P < 0.01), but these three were reduced after 72 hours as well (P ≤ 0.01). While the expression of VHL protein was increased at 24 hours, it was significantly reduced after 48 and 72 hours (P < 0.05). The protein expression levels of HIF-1α, HIF-1β, VEGF, and VHL at 72 hours were restored significantly after GAWD treatment in hypoxic condition (P < 0.01). The quantitative results are shown on Figures 9E–H, and the representative Western blots are shown in Figure 9I.

**TABLE 7:** Effect of high-altitude hypoxia on protein expression levels of HIF-1α, HIF-1β, VEGF, and VHL in MC3T3-E1 Cells ( $\bar{x}\pm s$ ).

Group	HIF-1α	HIF-1β	VEGF	VHL
Control	0.610 ± 0.067	0.578 ± 0.0167	0.619 ± 0.010	0.630 ± 0.072
High altitude 24 h	0.769 ± 0.053^##^	0.714 ± 0.053^##^	0.742 ± 0.024^##^	0.810 ± 0.048^##^
High altitude 48 h	0.835 ± 0.0457^##^	0.827 ± 0.0418^##^	0.901 ± 0.038^#^	0.495 ± 0.070^##^
High altitude 72 h	0.500 ± 0.083	0.4573 ± 0.054^##^	0.498 ± 0.081^##^	0.323 ± 0.080
GAWD + High altitude 72 h	0.9397 ± 0.0159^**^	0.947 ± 0.0516^**^	0.861 ± 0.0437^**^	0.977 ± 0.020^**^

Compared to the control group, the results indicate a statistically significant difference, with # denoting P < 0.05 and ## denoting P < 0.01. Additionally, when comparing the high altitude 72-h group, ** indicates P < 0.01.

## 4 Discussion

Both hypokalosis and hypoxia injury are associated with an enhanced osteoblastic injury. Here we demonstrate that the combined increase of simulated altitude and hypoxia exposure exacerbated the osteoblatic syndrome of hypoxia. The worst effect was seen in the condition of hypoxia at 4,500 m for 72 h, when cell viability was reduced to about 60%. In this case, a clear suppression of HIF-1α–VEGF signaling was seen, representative of a loss of the osteogenic and angiogenic function of HIF-1. At the transcriptional level, HIF-1α mRNA expression was temporarily increased under early exposure and subsequently reduced with extended exposure to hypoxia, indicating a dynamic feedback system. The balance of HIF-1α synthesis and degradation is critical to hypoxic adaptation. Moderate hypoxia kept homeostasis, while prolonged severe hypoxia disrupted the balance between HIF-1α synthesis and degradation, resulting in excessive HIF-1α accumulation with abnormal downstream activation, mitochondria functioning failure, and cell-cycle arrest. These data reveal dual protective and harmful functions of HIF-1 α – protective upon adaptive hypoxia but harmful when excessively activated during chronic high-altitude hypoxia. Treatment with GAWD significantly reduced the associated hypoxia-induced damage. After 48 h of GAWD under 4,500 m hypoxia, both mRNA and immunoprotein expression of HIF-1α, HIF-1β, VHL, and VEGF were significantly recovered comparing to those of the control cells. Correspondingly, accumulation of intracellular ROS and Ca^2+^ was alleviated, the mitochondrial membrane potential was partially restored, metastatic rates and G 1-phase arrest were significantly attenuated (P < 0.05). All of these findings suggest cytoprotective and antioxidative effects of GAWD, possibly by alleviating oxidative stress and restoring the balance of HIF-1 signaling under hypobaric hypoxia.

Past pharmacological investigations have shown that alkaloids derived from Aconites carmichaelii stimulate the energy metabolism of the mitochondrial system and the adaptive response via AMPK and NRf2 signaling, while phenolic compounds obtained from Zingiber officinale are potent ROS antioxidants and anti-inflammatory agents. Their administration together in GAWD may therefore have synergistic effects by simultaneously restoring mitochondrial efficiency and relieving oxidative stress. This complementary mechanism could account for the stabilisation of HIF-1α and restoration of VEGF expression recorded in prolonged hypoxia, however single-herb and coadministration combinations may need to be confirmed.

Taken together, the results of the current work point to the role of HIF-1-mediated angiogenic–osteogenic coupling in the High-altitude osteoporosis pathogenesis. As a key coordinator of angiogenesis, the HIF-1/VEGF axis not only facilitates neovascularization but also supports osteoblast differentiation and bone matrix constitution, thereby counterbalancing the process of bone resorption in the context of low oxygen. Our results suggest that GAWD possesses synergistic preventive and therapeutic effects in high-altitude-induced OP by repressing the HIF-1 pathway and restoring the function of osteoblasts.

Such mechanistic findings lend correlative support to TCM-based interventions for the management of OP in such high-altitude populations. However, our study did not involve safety or efficacy comparisons between GAWD and first-line Western anti-osteoporotic agents. Future work including animal toxicity testing and direct pharmacological comparisons was warranted to establish how safe and translational GAWD is. Furthermore, although our data revealed pronounced changes in the expression of HIF-1 α, VHL, and VEGF under simulated high-altitude hypoxia and in the presence of GAWD intervention, these connections remain correlative rather than causal. Direct manipulations of HIF-1 signaling (e.g., HIF-1 α inhibitor usage, siRNA knockdown, or chemical stabilizers) were not undertaken. Therefore, while our results highlight a potential role of the HIF-1 axis as a regulatory pathway in osteoblast injury and GAWD-related attenuation, functional studies are warranted to clarify the inducerality and to establish the precise molecular interactions implicated in the HIF-1/VEGF axis.

## 5 Conclusion

Here, we created a high-altitude hypoxia model in MC3T3-E1 osteoblastic cell line by exposing to 1,500 m atm for 72 h and found that 4,500 m atm treatment inhibited the expression of key regulatory proteins, such as Hification, HIF-1α, HIF-1β, VHL, and VEGF. This downregulation caused oxidative stress and Ca^2+^ accumulation, resulting in decreased cell proliferation and enhanced apoptosis, explaining the high-altitude–induced osteoblastic injury at the molecular level. The warming-Yang decoction with GAWD seemed to influence HIF-1 signaling axis, reduce ROS generation and intracellular Ca^2+^ accumulation, restore the function of mitochondria, and lessen morphology and function injury caused by hypoxia. Our results may provide the molecular basis for cytoprotective effect of GAWD and may provide potential therapeutic targets for the prevention and treatment of high-altitude–related osteoporosis. These results can provide an experimental basis for Further development of a new drug.

However, some limitations should be considered. The *in vitro* model cannot entirely mimic the complex physiological microenvironment *in vivo*, including variations in pH, nutrient supply, and metabolic activity, which may limit the generalizibility of the results. Additionally, our study mainly focused on the antioxidative effects of GAWD, and the causality of HIF-1 signaling was not assessed through genetic and/or pharmacological modulation, which remain to be explored in future studies. In addition, the combination of Aconitum carmichaelii and Zingiber officinale within the formula should be explored in future studies to confirm the purported synergistic mechanisms. Overall, these results require *in vitro* validation, pathway-level intervention, and individualized analyses of single-herb versus combined formulations to elucidate the mechanistic basis of cytoprotective effects.

## Data Availability

The data presented in the study are deposited in the figshare repository, available at: https://figshare.com/articles/dataset/Differential_gene_sequencing_data/30819326?file=60180644 (doi. 10.6084/m9.figshare.30819326).

## Abbreviations

GAWD, Aconitum carmichaelii and zingiber officinale decoction; ALP, Alkaline phosphatase; CCK-8, Cell counting kit-8; DEGs, Differentially expressed genes; HIF-1, Hypoxia-inducible factor-1; MMP, Mitochondrial membrane potential; OP, Osteoporosis; PHDs, Prolyl hydroxylases; RANKL, Receptor activator of nuclear factor κB ligand; ROS, Reactive oxygen species; SAL: Salidroside; TCM, Traditional chinese medicine; VEGF, Vascular endothelial growth factor; VHL, Von hippel–lindau tumor suppressor gene.
